# Clear cell renal cell carcinoma presenting as isolated symptomatic lumbar vertebral metastasis: a case report

**DOI:** 10.3389/fonc.2026.1853122

**Published:** 2026-06-30

**Authors:** Jinyan Wang, Qiaoqiao Jiang, Tong’en Chen

**Affiliations:** Department of General Practice, Ningbo No.2 Hospital, Wenzhou Medical University, Ningbo, Zhejiang, China

**Keywords:** bone metastasis, clear cell renal cell carcinoma, lumbar vertebra, multidisciplinary team, multimodal diagnosis, unknown primary tumor

## Abstract

We report a rare case of clear cell renal cell carcinoma (ccRCC) initially presenting as an isolated symptomatic lumbar vertebral metastasis. A 62-year-old man presented with progressive low back pain radiating to the right lower extremity. Elevated prostate-specific antigen (PSA) levels initially raised strong suspicion for prostate cancer with bone metastasis. However, comprehensive multimodal imaging combined with histopathological evaluation ultimately established the diagnosis of right-sided ccRCC with metastasis to the fourth lumbar vertebra (L4). This case highlights the critical importance of a systematic and multimodal diagnostic approach for bone lesions of unknown primary origin, even in the presence of misleading tumor-specific indicators. We further discuss the diagnostic challenges associated with occult RCC, key differential diagnoses, the role of multidisciplinary team (MDT) management, and current combined therapeutic strategies for metastatic RCC. Clear cell renal cell carcinoma, Bone metastasis, Lumbar vertebra, Unknown primary tumor, Multimodal diagnosis, Multidisciplinary team

## 1 Introduction

According to GLOBOCAN 2022 data, renal cancer ranks 14th in global incidence and 16th in cancer-related mortality (1). Renal cell carcinoma (RCC) accounts for approximately 90% of all renal malignancies, among which clear cell RCC (ccRCC) is the predominant histological subtype, comprising nearly 80% of adult renal cancers (2).In China, renal cancer remains a significant health burden, with an estimated 48,894 new cases and 16,179 deaths projected in 2024 (3).Currently, approximately 60% of RCC cases are incidentally detected through imaging. The classic triad—flank pain, hematuria, and abdominal mass—is now uncommon and typically associated with advanced disease (4). Although RCC frequently metastasizes to the lung, bone, lymph nodes, liver, and brain, bone involvement is usually multifocal (5).

In contrast, isolated symptomatic bone metastasis as the initial manifestation of an occult renal tumor is exceedingly rare and diagnostically challenging. Such atypical presentations often lead to delayed diagnosis or misdiagnosis. Herein, we present a rare case of ccRCC initially presenting as a solitary lumbar vertebral metastasis, along with a detailed analysis of the diagnostic pathway, multidisciplinary management, and therapeutic outcomes, supplemented by a focused literature review.

## 2 Case presentation

A 62-year-old male presented in October 2025 with a 2-month history of progressively worsening lumbogluteal pain radiating to the right lower extremity. The pain was described as dull and persistent, with partial relief upon ambulation. No numbness, hematuria, abdominal pain, or systemic symptoms were reported. Initial lumbar spine MRI revealed a space-occupying lesion involving the L4 vertebral body and right vertebral arch. The patient’s medical history was notable only for a prostatic nodule identified one year earlier without subsequent follow-up. Physical examination demonstrated localized tenderness over the L4 spinous process and paraspinal region, with no neurological deficits. Laboratory tests were largely unremarkable, except for elevated PSA levels (t-PSA: 10.040 ng/mL; c-PSA: 9.040 ng/mL). Routine chest computed tomography and abdominal ultrasonography were unremarkable. Lumbar spinal CT identified a space-occupying lesion highly suspicious for malignancy. Other tumor markers, including CEA and CA19-9, were within normal limits. Subsequently contrast-enhanced lumbar MRI demonstrated a space-occupying lesion in the L4 vertebral body and right vertebral arch with spinal stenosis, suggestive of a malignant tumor (**Figures 1A–C**).

**Figure 1:**
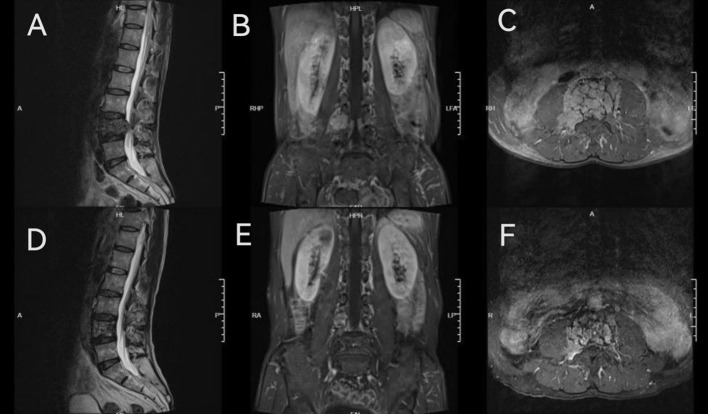
Pre-treatment contrast-enhanced lumbar MRI **(A)** sagittal T2WI, **(B)** coronal T1WI, **(C)** axial T1WI) demonstrated space-occupying lesions at the L4 vertebral body and right pedicle complicated by secondary spinal stenosis, measuring 74 × 43 mm at its largest extent (arrow). Post-treatment contrast-enhanced lumbar MRI after 7 cycles of therapy **(D)** sagittal T2WI, **(E)** coronal T1WI, **(F)** axial T1WI) demonstrated significant regression of the space-occupying lesions involving the L4 vertebral body and right pedicle, with improved secondary spinal stenosis (arrow).

Given the elevated PSA and previous abnormal prostate findings, prostate magnetic resonance imaging (MRI) with contrast enhancement was subsequently performed. The examination revealed benign prostatic hyperplasia with hyperplastic nodules in the transition zone, and the lesions were categorized as PI-RADS 2. Furthermore, whole-body positron emission tomography/computed tomography (PET/CT) revealed osteolytic destruction with increased metabolic activity in the L4 vertebral body and right pedicle, consistent with a malignant bone tumor (solitary plasmacytoma suspected). No other hypermetabolic primary or metastatic lesions were identified. Bone marrow examination was unremarkable. The patient was then transferred to the Department of Spine Surgery for CT-guided percutaneous needle biopsy of the L4 lesion. Notably, prior to the vertebral biopsy, the patient underwent renal ultrasound, chest CT and lumbar spine CT. No abnormal renal lesions were identified on all these imaging examinations. Histopathology combined with immunohistochemistry revealed positivity for PAX-8, CA9, CD10, and Vimentin, and negativity for CK7, Brachyury, TFE3, S-100, HMB45, and Melan-A, supporting the diagnosis of metastatic ccRCC (**Figure 2**).

**Figure 2:**
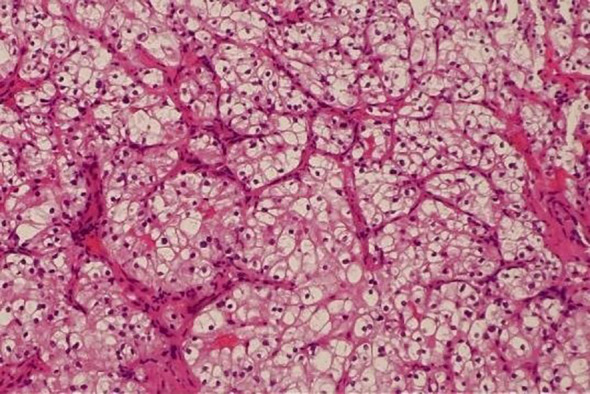
Hematoxylin and eosin (HE × 100) staining of vertebral tissue shows tumor cells with clear cytoplasm arranged in nests (arrow).

An MDT consultation was convened. Targeted contrast-enhanced renal MRI identified a 3.8 cm mass in the upper pole of the right kidney, exhibiting characteristic rapid contrast enhancement and washout, consistent with ccRCC (**Figure 3A**). The final diagnosis was right-sided ccRCC (cT1aN0M1, stage IV) with isolated L4 vertebral metastasis.

**Figure 3:**
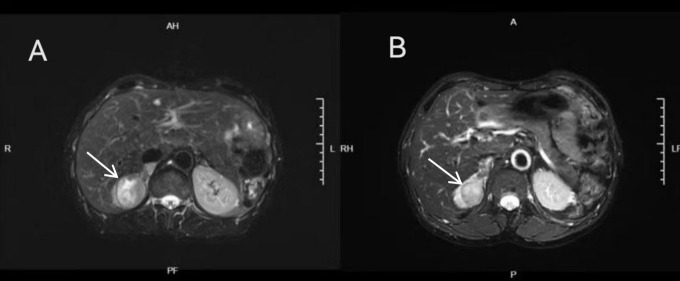
**(A)** Pre-treatment contrast-enhanced renal MRI demonstrates an enhancing mass in the upper pole of the right kidney measuring approximately 35 × 34 × 38 mm. (arrow). **(B)** MRI after 7 treatment cycles: A 24 × 27 × 38 mm tumor in the upper pole of the right kidney (arrow).

In accordance with the current first-line treatment guidelines for advanced clear cell renal cell carcinoma, a combined targeted and immunotherapeutic regimen was formulated following MDT discussion. The patient was treated with a combination of axitinib and toripalimab, along with denosumab for bone protection. Treatment was initiated uneventfully. After 7 cycles, follow-up contrast-enhanced MRI of the lumbar spine (**Figures 1D–F**) and kidneys (**Figure 3B**) demonstrated regression of both the primary renal lesion and the vertebral metastatic lesion. Notably, the patient’s lumbago was markedly relieved after 2 treatment cycles, and the PSA level remained stable throughout the treatment course.

## 3 Discussion

### 3.1 Diagnostic challenges in osteolytic lesions with misleading biomarkers

This case illustrates the diagnostic complexity of osteolytic lesions, particularly when tumor markers suggest an alternative primary origin. The elevated PSA level initially biased the diagnostic pathway toward prostate cancer, highlighting the risk of anchoring bias in clinical decision-making.

#### 3.1.1 Atypical presentation of RCC

Although the classic RCC triad is well known, it is now infrequently observed (4, 6). Approximately one-third of patients are diagnosed at an advanced stage with non-specific systemic symptoms or isolated metastases (4, 7, 8). Isolated bone metastasis as the first manifestation is rare and requires heightened clinical suspicion.

#### 3.1.2 Limitations of tumor markers

PSA elevation is not pathognomonic for prostate cancer and may lead to diagnostic misdirection. This case underscores that tumor markers should be interpreted cautiously and always in conjunction with imaging and histopathology.

#### 3.1.3 Limitations of PET/CT

PET/CT may fail to detect small or low-metabolic renal tumors. CcRCC often demonstrates low FDG uptake, and physiological renal tracer excretion can obscure lesions, leading to false-negative findings. Therefore, for patients with pathology confirmed as metastatic ccRCC, a negative PET/CT result should not exclude a primary renal origin. In retrospect, a contrast-enhanced thoracoabdominal CT would have been a more sensitive and cost-effective first-line screening tool for detecting occult RCC in a patient with an osteolytic lesion of unknown primary, given the characteristic ‘washout’ enhancement pattern of ccRCC. We recommend that clinicians prioritize contrast-enhanced CT over PET/CT for primary tumor screening in similar scenarios. PET/CT is not the initial diagnostic tool of choice but is primarily utilized for systemic staging, recurrence monitoring, and therapeutic response evaluation in advanced disease. In contrast, contrast-enhanced renal MRI provides superior sensitivity, particularly for small lesions, due to its ability to characterize vascular enhancement patterns. The MDT approach was pivotal in this case, integrating multidisciplinary expertise to refine the diagnostic strategy and identify the occult primary tumor efficiently.

### 3.2 Critical role of histopathology

Biopsy with immunohistochemical analysis remains the gold standard for diagnosing metastatic tumors of unknown origin. The IHC panel in this case was decisive:PAX-8: A highly sensitive and specific marker for renal and Müllerian system differentiation, positive in approximately 95% of cases (9).CA9: Carbonic Anhydrase IX, a downstream effector of the VHL pathway, is pathognomonic for ccRCC, expressed in 75% to 100% of tumors (9).CD10 and Vimentin: Common positive markers in ccRCC.CK7 (–): Helps exclude other renal tumors such as urothelial carcinoma. Negative markers (Brachyury, TFE3, S-100, HMB45, Melan-A): Rule out chordoma, TFE3-rearranged RCC, and other mesenchymal/melanocytic lesions. This immunoprofile was decisive in establishing the diagnosis and guiding further investigation.

### 3.3 Therapeutic strategies

The treatment landscape for metastatic ccRCC has evolved toward combination regimens of targeted therapy and immune checkpoint inhibitors (10–13).Following establishment of the diagnosis, the patient was transferred to the Department of Medical Oncology to receive targeted therapy combined with immunotherapy consisting of axitinib plus toripalimab.

The RENOTORCH study demonstrated that first-line toripalimab plus axitinib significantly improved progression-free survival (PFS) and objective response rate (ORR) compared to sunitinib, with a trend toward overall survival (OS) benefit, especially in patients with intermediate/poor risk disease (14, 15). This regimen is approved by the National Medical Products Administration (NMPA) of China and recommended as first-line therapy for intermediate/poor risk metastatic RCC (mRCC) in authoritative guidelines, including the CSCO Renal Carcinoma Guidelines (usually as a Category I recommendation) (10).

In this patient, significant pain relief was observed after 2 cycles of therapy, and follow-up contrast-enhanced MRI after 7 cycles confirmed tumor regression. The role of local therapy for isolated bone metastasis remains individualized. Bone-modifying agents such as denosumab are essential to reduce skeletal-related events.

## 4 Conclusion

This case provides several important clinical insights:

1. Isolated bone lesions require a broad differential diagnosis, regardless of suggestive tumor markers.
2. A systematic diagnostic strategy integrating imaging and pathology is essential for identifying occult primary tumors.
3. CcRCC should be considered in cases of unexplained osteolytic spinal lesions, even in the absence of urinary tract symptom.
4. MDT-based management is critical for optimizing diagnosis and treatment in complex oncological cases.

This case report has inevitable limitations. Based on a single patient, we cannot make inferences regarding causality or disease prevalence. More clinical data from larger cohorts are required to address these issues in the future.

## Data Availability

The original contributions presented in the study are included in the article/supplementary material. Further inquiries can be directed to the corresponding author.
